# Structure, space and size: competing drivers of variation in urban and rural measles transmission

**DOI:** 10.1098/rsif.2020.0010

**Published:** 2020-07-08

**Authors:** Hannah Korevaar, C. Jessica Metcalf, Bryan T. Grenfell

**Affiliations:** 1 Office of Population Research, Princeton University, Princeton, NJ, USA; 2 Woodrow Wilson School of Public and International Affairs, Princeton University, Princeton, NJ, USA; 3 Ecology and Evolutionary Biology, Princeton University, Princeton, NJ, USA; 4 Fogarty International Center, National Institutes of Health, Bethesda, MD, USA

**Keywords:** measles, urban, rural, principal components analysis, disease transmission

## Abstract

A key concern in public health is whether disparities exist between urban and rural areas. One dimension of potential variation is the transmission of infectious diseases. In addition to potential differences between urban and rural local dynamics, the question of whether urban and rural areas participate equally in national dynamics remains unanswered. Specifically, urban and rural areas may diverge in local transmission as well as spatial connectivity, and thus risk for receiving imported cases. Finally, the potential confounding relationship of spatial proximity with size and urban/rural district type has not been addressed by previous research. It is rare to have sufficient data to explore these questions thoroughly. We use exhaustive weekly case reports of measles in 954 urban and 468 rural districts of the UK (1944–1965) to compare both local disease dynamics as well as regional transmission. We employ the time-series susceptible–infected–recovered model to estimate disease transmission, epidemic severity, seasonality and import dependence. Congruent with past results, we observe a clear dependence on population size for the majority of these measures. We use a matched-pair strategy to compare proximate urban and rural districts and control for possible spatial confounders. This analytical strategy reveals a modest difference between urban and rural areas. Rural areas tend to be characterized by more frequent, smaller outbreaks compared to urban counterparts. The magnitude of the difference is slight and the results primarily reinforce the importance of population size, both in terms of local and regional transmission. In sum, urban and rural areas demonstrate remarkable epidemiological similarity in this recent UK context.

## 1. Introduction

Though widespread vaccination has greatly reduced global transmission of measles since the mid-1960s, it continues to be a major cause of death among children in sub-Saharan Africa [1]. Additionally, re-emergence of measles in many parts of the world due in part to vaccine hesitancy emphasizes the importance of continued attention to measles [2,3]. More broadly, in an increasingly urbanized world, understanding the impacts of urbanization and population density on transmission of highly contagious infections such as measles are increasingly urgent [4]. A simple epidemic clockwork and detailed and reliable notification systems across urban and rural settings, makes measles one of the best documented spatio-temporal consumer–resource model systems generally, and a particularly apt candidate for examining disease interactions across diverse population densities [5–8].

Urban and rural disparities in health have been studied in a variety of contexts. In the USA, the urban–rural gap in life expectancy has widened over the last four decades. This is due in large part to mortality of individuals under the age of 25 and correlates with accidental injury and reduced access to high-quality medical care [9,10]. The incidence of Dengue is known to be higher in urban areas [11,12], whereas the burden of malaria is higher in rural areas [13]. In rural areas, increased likelihood of concurrent sexual contacts increases the risk for HIV and other STIs [14]. Seasonal migration between urban and rural areas also impacts transmission of infection [15]. While these papers have investigated differences in urban and rural health outcomes, disease burden, and migration few have explored the urban/rural hierarchy in transmission across a metapopulation or attempted to quantify differences in transmission due to urban/rural environment. This study allows an investigation into differences within urban and rural areas in addition to differences between urban and rural areas as part of a larger, connected population.

Ferrari *et al.* show that population size is the most consistent driver of the magnitude of epidemic seasonality in measles epidemics in Niger across urban and rural districts [16]. Rainfall and agriculturally induced variation in contact rates also impact the amplitude of seasonality. The authors find reduced seasonal forcing in sparsely populated areas with the highest seasonal amplitudes present in large and/or densely populated areas. A comparable investigation of urban and rural districts in a context where school calendar forcing is the dominant mechanism has not been done. The consistency of seasonality in England and Wales (hereafter E&W) as a result of the school calendar provides a unique opportunity to compare transmission rates and epidemic dynamics while isolating urban/rural status from other potentially confounding factors such as climate, variation in seasonal contact rates, population size and proximity to epidemic pacemakers. Additionally, the granularity of the data as a result of the number of districts (1422) and the duration of notification data before vaccination (20 years) is unparalleled.

### 1.1. Time-series susceptible–infected–recovered model

Measles is a paradigmatic infection for investigating nonlinear dynamics of disease transmission [17–19]. Infection with strongly immunizing pathogens such as measles results in either death of the host, or more often, recovery and lifetime protection. Compartmental models, such as the susceptible–infected–recovered (SIR) model are useful as simple models of disease dynamics but can be difficult to adapt to data. The SIR model assumes a well-mixed population, and in the most basic form balances demographic processes (e.g. births, deaths and immigration) with properties such as contact rate, *β*, and infectious period particular to a given pathogen. In general, the transmission coefficient, *β*, varies seasonally, in the case of measles, this seasonality is largely driven by the school calendar.

While the simplicity of the SIR model is beneficial for interpretability and cross-setting comparisons of transmission, calibrating the seasonality-forced SIR model against data can be statistically challenging [6,20]. The main challenges of fitting the SIR model to data result from two sources: only one state variable is observed (the number of cases) and rates of under-reporting are not known. A computationally efficient option for addressing both these challenges is the time-series SIR (TSIR) model. The TSIR model relies on two main assumptions: first, the infectious period is fixed at the sampling interval of the data (e.g. biweekly for measles) and that over a long enough time (e.g. 10–20 years), the sum of births and cases should be approximately equal due to the high infectivity of pathogens such as measles and other childhood infections. Both of these assumptions have been thoroughly tested and found to be largely appropriate for this pre-vaccine era data [19]. Fixing the infectious period to be equal to the sampling period means we assume an individual that is infected at the *n* time step will be recovered by *n* + 1.

### 1.2. Metapopulation dynamics and spatial coupling

Highly transmissible childhood infections, such as measles, can spread quickly through a community until the susceptible population is depleted, to the point that it can lead to local extinction. Thus measles requires a steady stream of new susceptible hosts (mainly from births) to remain endemic. For this reason, large populations, typically above 300 000, are required for sustained transmission—this threshold is called the critical community size (CCS). For communities below the CCS, future outbreaks are dependent on imported infections from other locations [21]. In these small populations, the susceptible proportion will increase until the pathogen is reintroduced through spatial transmission from a neighbouring community. These metapopulation dynamics, the reintroduction of infections via spatial contacts, are a characteristic component of measles transmission in E&W during pre-vaccination years (1944–1965) [5,17,22]. Endemic areas such as London would act as epidemic pacemakers, replenishing infections for communities below the CCS threshold. Echos of London’s strong biennial epidemic pattern radiate across the surrounding region, creating biennial epidemics in locations that would otherwise be too small to experience such regular outbreaks [5]. Quantifying rates of spatial transmission is a crucial challenge for epidemiologists as the spread and persistence of pathogens depends on this connectivity between large and small places [18,22–24].

Population gravity and spatial hazard models have been used to estimate movement of individuals across locations [22–25]. Population gravity models assume that movement between locations can be approximated by the product of the population size normalized by a transformation of the distance between them. Under these assumptions, we expect movement between large places to be high, movement between small places to be low, and for contact between communities to decline with distance. These models have been able to successfully capture population movement in the context of disease transmission in some cases [25]. Though gravity models have provided useful insights into the spatial interactions of many human and non-human disease systems, in the absence of independent covariates describing human movement, the simultaneous inference of epidemic trajectories and spatial coupling is difficult. Information on the movement of school age children is notably sparse. As school age children are the population of interest in this case, we opt to avoid weighting by population and allow the reconstructed susceptible population to guide our probabilistic model. Furthermore, research has shown that for this dataset in particular, gravity models have not adequately captured the dynamics of large cities with off-year peaks or coastal cities [22,25]. Finally, gravity models do not assume different contact rates by urban or rural location type and we may expect contact between urban locations to be higher regardless of population size. For these reasons, we use a spatial hazard model to calculate the spatial coupling associated with each location. Resulting estimates are informed by the measles case data (using susceptible and infectious dynamics to determine infection probabilities) and assume no predetermined functional forms, so that estimates of coupling for each location are not constrained by location size or distance [24].

Spatial coupling provides an estimate of how much a district’s epidemic dynamics are influenced by the influx of new cases from other locations. Previous hazard-based coupling estimates suggests that transmission across locations is strongly correlated with population size [24]. Bjornstad *et al*. use a spatial hazard model to estimate spatial coupling for all 954 urban areas in the dataset. The authors show larger places exhibit larger coefficients of spatial coupling than smaller, more isolated places, and thus more coordination with national epidemics. Additionally, the authors use residuals from linear regression of spatial coupling on population size to show that locations near large endemic locations (such as London, Manchester and Birmingham) have higher than expected estimated rates of coupling than other locations of comparable size. Similarly, locations very far from these population centres produce lower than expected coupling rates. This highlights the importance of both size and space in cross-location measles transmission.

By contrast, research on data from E&W suggests transmission within locations does not scale with location size. Bjornstad *et al.* [26] use a subset of sixty cities in E&W to show that while transmission rates demonstrate some variability across locations, they do not vary uniformly with population size. The authors select 60 locations of various sizes and calculate the basic reproduction number based on the epidemic data. The basic reproduction number (*R*_0_) is a parameter commonly used to quantify the contagious power of disease, it is defined as the number of secondary infections resulting from a single infected individual if everyone else in the population is susceptible. Population level estimates of *R*_0_ for measles are commonly between 18 and 30 [27]. Though the estimate can vary, Bjornstad *et al.* posit that it does not vary systematically by population size. This is likely because schools act as transmission hotspots and the importance of these focus points outweighs any impact of population size. Furthermore, measles has a particularly high transmission rate: infected individuals are contagious for up to 4 days before they show symptoms and the measles virus is airborne and can survive up to 2 hours in airspace. These factors make the disease highly contagious. For this reason, once an infection is introduced to a susceptible population it will spread rapidly. As these infections will largely spread in schools, any differences due to population density are believed to be marginal, particularly in the case of E&W [26].

### 1.3. Potential urban and rural differences

Estimates of transmission allow us to measure within-community epidemic dynamics. Spatial coupling allows us to estimate how these locations differ in their connection to metapopulation dynamics. We can, therefore, measure potential differences between urban and rural locations locally as well as contextually. However, we know spatial proximity plays a key role, both in terms of the local population dynamics as well as the number of imports a location can expect to receive. We also know urban and rural areas are not distributed randomly in space (both rural and small population locations are more likely to be farther from large urban areas) this indicates a need to control for spatial effects.

Though the relationship between population size and measles transmission has been the subject of many studies, previous E&W work has only focused on analysing data from urban districts, leaving a rich dataset of 468 rural districts almost entirely untouched.

We examine both spatial connectivity and within city measles transmission in urban and rural areas in 1422 locations in E&W. In a 1998 paper, Bolker & Grenfell examine the aggregate differences between urban and rural districts, using a subset of 1302 of these locations [28]. Much of the analysis in the 1998 paper examines the aggregate dynamics, combining case data from all urban and rural locations to compare timing and epidemic intensity. Furthermore, the authors do not estimate epidemic parameters (such as transmission and susceptible population) from the data but rather compare aggregate urban and rural trends to an urban–rural patch model. These authors leave the question of spatial diffusion between urban and rural locations largely unanswered. Furthermore, the authors note a strong spatial correlation across the E&W metapopulation and highlight the necessity of investigating these spatial patterns in greater detail [19]. This paper investigates these differences at the individual district level and probes urban/rural differences at a fine spatial scale.

We may expect to see differences in the disease ecology of urban and rural locations for several reasons. It is possible that the decreased population density in rural areas leads to a fewer contacts within these locations, resulting in slower transmission of measles in rural areas relative to their urban counterparts. Variation in birth rates between urban and rural locations may impact transmission by replenishing the pool of susceptible individuals at different rates.

Differences in the number or size of schools—the primary location of outbreaks—may also impact the transmission of the disease. As susceptible contacts are generally driven by the school calendar, measles transmission in E&W typically has a consistent seasonality. We see peaks in transmission when students return from holidays, when susceptible populations are at their highest and when susceptible individuals are coming into frequent contact. If there are differences in the spatial proximity of schools we may see different transmission rates or different outbreak patterns. In particular, when schools are farther apart and mixing between them is relatively weak, we might observe either multiple small epidemics or slower progression of the disease through the district [16,17,29,30].

Finally, if the migration or mobility of individuals occurs at uneven rates between location types, this may impact the probability of introducing new infections, and therefore spatial coupling estimates. If population movement between locations depends on more than just population size we may see differential case import frequency between urban and rural areas. For example, people may move between urban locations with more frequency than from urban to rural or between rural locations, in which case we will see lower estimates of spatial coupling in rural locations than in urban locations. We may expect urban to urban travel to be more common than urban to rural travel regardless of population size. For example, in the USA, COVID-19 has been comparatively slow to spread to rural areas even as cases skyrocketed in urban areas [31].

Using estimates of transmission rates and spatial coupling, we compare all urban and rural districts. At the aggregate, we find a surprising amount of coherence: both internal dynamics and spatial coupling show consistent dependence on population size. We further restrict our sample to neighbouring urban and rural districts to isolate the potentially confounding associations between size, location and urban/rural designation. We find that size is consistently a more significant driver of epidemic dynamics than location type. The exchange of outbreaks between neighbours is dependent on population size, with larger locations frequently introducing outbreaks to their smaller neighbours. In this way, these mini-communities mirror national metapopulation epidemic cascades. However, we do find slight distinctions in the epidemic behaviour of urban and rural areas, namely that rural areas are characterized by more frequent outbreaks which infect fewer individuals. This suggests rural areas may sustain epidemics through internal rescue effects [23], and highlights the importance of accounting for heterogeneous mixing patterns to uncover subtle differences in epidemic spillovers.

## 2. Data

To explore differences in urban and rural areas, we analysed pre-vaccination weekly measles incidence data from 1944 to 1965 E&W [5,32]. This dataset is unusually rich, with 954 urban cities and towns and 457 rural districts. In addition, we used annual births, population and geographical location of each district. For the paired analysis, we use 179 urban districts with a rural neighbouring district (for a total of 358 districts).

The classification of districts as urban or rural was not strictly scientific at this time. The system of the era involved a combination of considerations such as population density (measured in people per acre), level of urban development, and the type of local government (e.g. urban council or parish) [33]. Historical documentation indicates that this system of classification was at times arbitrary and resulted in a misclassification rate of approximately 20% according to contemporary standards [33]. Still, this classification is a feature of the dataset and likely represents some amount of structural difference between locations. Additionally, if the misclassification occurred at random or resulted in more frequent classification of small sparsely populated districts as urban this would attenuate any differences we detect. Additionally, we believe our strategy of selecting urban/rural districts will mitigate any potential misclassifications. As these are neighbouring districts with the same name that have been distinguished from each other by ‘urban’ and ‘rural’ labels, we expect the urban districts to be at least more dense than their rural neighbours, even if they are not dense in a global sense. In other words, though it is unlikely these data capture the global range of population density and sparsity, we do expect that neighbours will differ from each other. In fact, we were able to obtain land area for 136 of the 179 rural districts used in the paired analysis, and 78 of the urban districts (73 pairwise complete). We obtained these estimates from the Wellcome Trust (UK Medical Heritage Archive); major boundary changes in 1972 necessitate obtaining land area estimates contemporary with the case data. For this subset of districts, the rural areas are consistently lower density (electronic supplementary material, figure S5). The least dense district is approximately 0.03 people per acre, and the most dense is about 28 people per acre. While these do not represent global extremes of population density/sparsity, they provide enough variation to explore measles dynamics under different density conditions.

Incidence data were aggregated to the biweekly scale for modelling analysis (described below). The diversity of locations in terms of geographical space and population size, as well as the temporal detail of the incidence data provide an unparalleled and uniquely apt dataset for investigating urban and rural differences in transmission. The 20-year epoch covered by the data allows for a robust study of outbreaks as well as sufficient opportunities to compare urban and rural epidemics. Furthermore, pre-vaccination data allow us to understand transmission patterns without uncertainty related to vaccination coverage, this provides the most direct estimates of transmission rates and mixing dynamics. The urban data used in this paper have recently been made available [32], the rural data and an R studio notebook to replicate the results of this paper are available in the electronic supplementary material. Much of the analysis on the disease dynamics is done using the open source R package: tsiR [34].

## 3. Methods

### 3.1. The time-series susceptible–infected–recovered model

We compared population dynamics and transmission within urban and rural using a number of metrics. We first examined epidemic fadeouts (time between epidemics) to see if urban and rural areas differ in the pathogen extinction rates. As these estimates may be subject to bias due to systematic differences in reporting rates, we also calculated the number and length of three-week fadeout which previous research has shown to be robust to under-reporting [26]. We also computed average birth rates as well as the coefficient of variation in births. Births may impact disease dynamics by altering the yearly influx of susceptible individuals. Finally, directly from the incidence data, we calculated epidemic growth rates which we expect may correspond to differences in population mixing. To further assess local disease dynamics, we use a TSIR model [19,26,35] to obtain estimates of seasonal transmission. The TSIR model is a discrete time mechanistic model where the susceptible dynamics can be modelled as

3.1 $$S_{t+1}=B_{t}+S_{t}-I_{t+1}.$$

The susceptibles at *t* + 1 (*S*_*t*+1_) are simply the previous susceptibles (*S*_*t*_) plus births (*B*_*t*_) minus the new infections (*I*_*t*+1_). The associated deterministic infected dynamics are

3.2 $$I_{t+1}=\beta_{t}S_{t}I_{t}^{\alpha }.$$

The seasonally varying transmission rate is estimated as *β*. The tuning parameter, *α* acts as a correction factor for moving from discrete to continuous time [19]. In a purely theoretical sense *α* should be equal to unity in continuous time [20], however, discretized models produce more accurate predictions with *α* values slightly under unity [26]. To be consistent with previous estimates for this dataset, we fixed *α* to be 0.97 [19,26].

The primary assumption of the TSIR model is over a sufficient period of time, due to the high transmission rate of measles, everyone should acquire the infection. This allows us to assume that cumulative cases and cumulative births will be approximately equal, yielding an estimation of reporting rate. We can then reconstruct the susceptible population at each time step. With estimates of both the infected (reported cases divided by reporting rate) and susceptible dynamics, equation (3.2) can be log-transformed into a linear model

3.3 $$\log[I_{t}+1]=\log\beta_{t}+\log S_{t}+\alpha \log I_{t}.$$

From equation (3.3), we can estimate both the seasonal transmission rate (*β*_*t*_) and an approximate measure of *R*_0_ (=*β*_*t*_*N*, where *N* is mean population size). We can also evaluate the seasonality by calculating the coefficient of variation in *β*_*t*_, this allows us to measure whether transmission is variable over the year or relatively constant [29,35,36]. A full discussion of the implementation of TSIR can be found in [6,35,36]. A discussion of result sensitivity to estimation procedure (such as the regression type selected for susceptible reconstruction) can be found in the electronic supplementary material. Additional information on model fit and parameter estimates across locations can also be found in the electronic supplementary material.

### 3.2. Epidemic coupling

The primary dynamic exchange of interest in this section is contact between susceptible individuals in a single district (local) with an infected individual from another district (regional), and whether such contact sparks an epidemic in the susceptible’s district. In line with previous analysis, we include all other districts as regional, and thus we are estimating the epidemic coupling between one community and all other communities in our dataset [24]. We use the reconstructed susceptible dynamics as well as estimates of *β* to calculate epidemiological coupling for each location. Following extinction, the local dynamics are converted into a waiting time distribution, for which the probability that a fadeout will end is governed by the probability of contact between local susceptibles and regional infectives as well as the probability that an epidemic will result from such contact. Spatial contact depends on the probability that a local individual is susceptible, the probability a regional individual is infected, and the spatial isolation of the local community (1/*c*_*j*_, where *c*_*j*_ is the coefficient of coupling).

We want to estimate the probability that contact occurs and that an epidemic is sparked. In other words: P(A∩B)=P(A|B)P(B). Here, *A* is the probability of an epidemic occurring and *B* is the probability of contact between a local susceptible individual and a regional infected individual. In order to estimate the probability of an epidemic, we estimate the number of susceptible individuals at each time step as in equation (3.1).

We use a modified version of equation (3.2) in which the expected number of infections is given by

3.4 $$\lambda_{t,j}=\beta_{u,j}{\left(I_{t,j}+i_{t,j}\right)}^{\alpha }S_{t,j}.$$

*I* are local infected individuals and *i* are infections arising from regional contact, and *β*_*u*_ is the seasonal transmission rate which corresponds to *t*. We expect *β* to vary within the year but to be relatively consistent across years for a single location *j*, thus it will fluctuate according to a biweekly indicator *u*, rather than continuously over time. We can model the trajectory of the epidemic as a piecewise-constant (at the scale of a single generation) birth–death process [21]. If we assume a *per capita* birth rate in infections, in this case *λ*/(*I* + *i*), then starting with one infected individual the number of infected individuals in the following generation will be distributed according to a geometric distribution with expectation *λ*/(*I* + *i*). Beginning with *I* + *i* infected individuals, we get a sum of *I* + *i* geometrics, it follows that the distribution of infections at *t* + 1 as a function of infections at *t* is

3.5 $$I_{t+1,j}∼\text{NegBin}(\lambda_{t,j},I_{t,j}+i_{t,j}).$$

NegBin signifies a negative binomial process with expected value *λ*_*t*,*j*_, and a clumping parameter *I*_*t*,*j*_ + *i*_*t*,*j*_. The probability of spatial contact between a local susceptible and a non-local infectious individual is modelled as

3.6 $$1-\exp(-c_{j}x_{t,j}{\bar{y}}_{t,k\neq j}).$$

The proportion of *local* susceptibles is *x*_*t*,*j*_ and ${\bar{y}}_{t,k\neq j}$ is the proportion of infectious *non-locals*, that is, the proportion of infectious individuals across all districts *k* which are not district *j* at time *t*. Note that *x*_*j*,*t*_ corresponds to proportions of susceptibles (*S*_*j*,*t*_/*N*_*j*,*t*_), and ${\bar{y}}_{t,k\neq j}$ is the proportion of infectious individuals ($\sum_{k}I_{k\neq j,t}/\sum_{k}N_{k\neq j,t}$). Finally, *c*_*j*_ is the coupling coefficient of location *j*. This coupling measure is analogous to other variants, such as the coupling coefficient of the population gravity model [25]; however, we use this non-parametric (with respect to coupling) version so our estimation procedure (as follows) can be guided by the epidemic data itself while minimizing *a priori* assumptions regarding population movement.

Given contact has occurred, the probability that an epidemic does not occur is given by 1/(1 + *β*_*t*,*j*_*S*_*t*,*j*_), this is given by the null probability of the negative binomial distribution (equation (3.5)) when *I* = 0 and *i* = 1. An epidemic will occur by the complementary probability

3.7 $$1-\frac{1}{1+\beta_{t,j}S_{t,j}}=\frac{1+\beta_{t,j}S_{t,j}-1}{1+\beta_{t,j}S_{t,j}}=\frac{\beta_{t,j}S_{t,j}}{1+\beta_{t,j}S_{t,j}}.$$

Putting together the probability of spatial contact and the probability of an epidemic we obtain the discrete-time hazard

3.8 $$(A\cap B)=P(A|B)P(B)\to h(t,j)=\frac{\beta_{t,j}S_{t,j}(1-\exp(-c_{j}x_{t,j}{\bar{y}}_{t,k\neq j}))}{1+\beta_{t,j}S_{t,j}}.$$

This is an increasing function with the number of local susceptibles and the proportion of non-local individuals that are infectious, it may change with population size if isolation is size-dependent [24]. Conditional on the local susceptible population and regional prevalence of infection, the theoretical waiting time distribution can be written as the expectation of a binomial process for which the log-likelihood of the fadeout is given by

3.9 $$l(c_{j}|I_{t-1,j}=0)=\sum_{}ln(h_{t,j}^{z_{t,j}}{\left(1-h_{t,j}\right)}^{1-z_{t,j}}).$$

The binary indicator *z*_*t*,*j*_ is equal to 1 if *I*_*t*,*j*_ > 0 and equal to zero otherwise, and *h*_*t*,*j*_ is given by equation (3.8). We sum over all observations for which the local infections remain at 0, up until an outbreak occurs. This allows us to calibrate *c*_*j*_ by the moment an epidemic is sparked. This is an adaptation of the typical binomial likelihood function with the probability of an outbreak determined by *h*_*t*,*j*_ (and thus the probability of no outbreak is (1 − *h*_*t*,*j*_)). We sum over all observed outbreaks for each location and use Newton–Raphson maximum-likelihood estimation to obtain an estimate of *c*_*j*_ from equation (3.9). Note that it is not possible to estimate *c* for those communities in which measles is endemic (i.e. there are no fadeouts). There were 29 communities (out of 1422) that did not have a sufficient number of fadeouts to estimate the coupling coefficient, these locations are dropped from the coupling analysis but are included in the comparison of other measures of epidemic behaviour.

In keeping with previous findings [24], we expect to see high coefficients of coupling for locations below the critical community threshold that are geographically proximate to larger districts which provide the reintroduction of pathogens. We expect small isolated areas to have the lowest estimates of coupling because imported infections as a result of human mobility will be relatively rare and thus the reintroduction of pathogens will have correspondingly lower probability.

To illustrate the importance of spatial proximity more explicitly, we calculate the correlogram of incidence data across the entire dataset. This estimates the spatio-temporal correlation of the incidence data and its dependence on distance. We also examine districts within 150 km of London. We calculate the correlation of incidence data for each of these districts relative to London and use a generalized linear model to estimate the association of distance, population size and district type on similarity to London’s case data.

### 3.3. Principal components analysis on paired data

Probing differences further, we then subsetted our analysis to neighbouring urban and rural districts to isolate space from size and location type. These districts are adjacent and non-overlapping such that they allow us to control for spatial proximity and measure the relative influence of urban versus rural status and population size. These districts are sampled from a variety of spatial locations across E&W so the results are not a feature of a single area.

We selected a representative sample of 179 pairs (a total of 358 districts) below the CCS and used principal components analysis (PCA) to uncover the correlations between demographic characteristics (birth rates) and estimated parameters (susceptible fluctuations, transmission rates) and subsequently to see how urban and rural areas vary across these numbers. PCA is the eigenvalue decomposition of the covariance matrix of scaled covariates. We scale the data so each variable column has zero mean and unit variance, this ensures variables with larger values are not given greater weight due to their higher variance. Eigenvalue decomposition factorizes a matrix into its canonical form. It produces the vectors that (ranked by their eigenvalues) explain the most variance within the data. PCA uses an orthogonal transformation to project a matrix possibly correlated covariates onto a new uncorrelated basis space. PCA has been used to identify the genes that are responsible for the most population-level variation populations [37] as well as to isolate dominant frequencies in complex signals [38].

This method demonstrates (1) how variables are related to each other, (2) which variables are most influential in terms of looking for differences in the data and (3) whether population size or urban/rural designation influence how locations score on these maximal variance vectors. PCA enables an investigation of multiple variables simultaneously as well as isolating the importance of variables rather than testing each covariate separately. We withhold urban and rural indicators as well as population size so we can test their influence on the projections. After obtaining our principle components (eigenvectors), we project each location onto the first two principal components (the vectors responsible for the two dimensions of most variance). We compare each city’s score with its rural neighbour to assess the influence of space. If space is the primary driver, we expect each location to be similar to its neighbour. We also compare the scores by population size. Finally, we calculate the euclidean difference between each pair, this gives us a measure of how different each location is from its neighbour across the dimensions of highest variance. We then check the association of this distance with their difference in size. These comparisons together demonstrate the comparative influence of space, size and environment.

### 3.4. Epidemic exchange in paired locations

We further attempt to disentangle the importance of district type by investigating the timing and duration of epidemics between pairs. Many of the paired districts are small (median population 15 000; range 700–250 000), and fitting the TSIR model to locations can be challenging due to frequent and lengthy extinctions. Making comparisons directly from the time series enables us to concretely measure timing and coordination of epidemics to assess how and if districts interact with each other. In particular, we evaluate the proportion of rural epidemics which occur during a simultaneous epidemic at its urban neighbour. Similarly, we assess which member of each pair leads or lags in local epidemics. For each pair of districts, we evaluate the proportion of its epidemics which are preceded by an epidemic in it is neighbouring district. These proportions provide a measurement of how many epidemics can be attributed to the urban or rural component of each pair of districts. We also compare the total number of outbreaks and the number of large epidemics (greater than 14 weeks) between pairs.

## 4. Results

### 4.1. Aggregate urban and rural comparison

**Figure 1.**
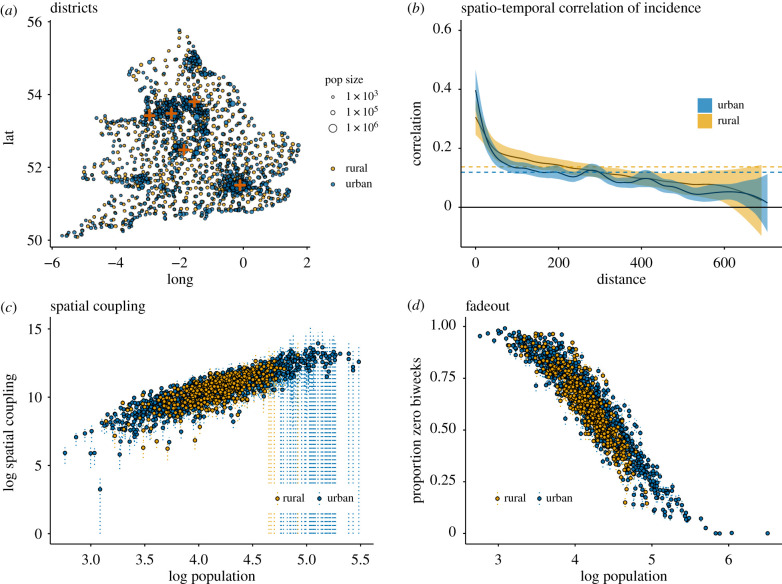
Epidemic coupling and outbreak dynamics in urban and rural E&W 1944–1965. (*a*) Plots the longitude and latitude of each of the 1422 locations colour coded by urban–rural status and scaled by mean population size. The five largest locations (London, Birmingham, Manchester, Liverpool and Leeds) are marked by red crosses. (*b*) Patterns of measles incidence show strong spatio-temporal correlation across E&W with a strong dependence on distance. While correlation of outbreaks is high among near neighbours, this correlation decreases rapidly with distance until about 200 km where it slowly falls below the average temporal correlation for both urban (0.12) and rural (0.14) districts. The spatial correlation of all locations combined is consistent with these separate estimates. (*c*) Analysing coupling as a function of population yields a strong log-log linear relationship across both urban and rural cities. This is consistent with previous findings we do not see significant differences between urban and rural areas controlling for population size. At the large populations, we see a large increase in the size of confidence intervals. This is due to the small number of interepidemic periods in large locations. With few opportunities to calculate coupling, the standard errors increase drastically. (*d*) Similarly, we find strong agreement between the urban and rural relationships for fadeouts and population. These figures demonstrate the importance of population size as well as proximity in determining epidemic dynamics.

Investigating aggregate differences, such as epidemiological spatial coupling and fadeouts, between urban and rural districts, we find a consistent relationship with population size but no obvious difference by urban or rural designation. These findings are consistent with previous estimates for urban districts [24,28]. Coupling increases log-linearly with population size for both urban and rural areas indicating that imports increase with population size (figure 1). The analytical relationship between fadeouts, coupling, and population size is thoroughly investigated in Bjornstad *et al’s* 2008 paper [24]. As population size increases, so too does the susceptible class, this increases sensitivity to imports and increases the probability of an epidemic. Probabilistically, this increased sensitivity leads to fewer ‘missed’ epidemics and consequently reduces the proportion of fadeouts, even if import rates are consistent across locations. We are primarily interested to see if the advantages associated with increasing population size are different for urban and rural areas. Figure 1 would suggest the returns to population size are consistent. However, we know that the opportunity for imports also increases with proximity to endemic locations so to isolate the impact of urban and rural designation, we need to further control for this proximity.

Additional comparisons of TSIR parameters such as transmission rate (*β*) and *R*_0_ reveal variation with population size, but urban and rural locations remain consistent (electronic supplementary material, figure S5). The proportion of biweeks without cases correspondingly decreases with population size for both urban and rural areas. However, figure 1 further demonstrates the importance of spatial proximity to large metropolitan areas such as London, Birmingham, Manchester, Liverpool and Leeds in terms of both urban/rural designation as well as population size.

Figure 1*b* shows the spatio-temporal correlation of incidence in urban and rural areas across the entire dataset. We see that correlation decreases with distance for both urban and rural areas, though the urban decline is more precipitous. The spatial correlation of population size is approximately 0.13 for near neighbours but it falls below to statistically zero after about 40 km. It is likely that population scaling as well as urban/rural distinction is a factor in this correlation. Locally, population is spatially correlated; at the national scale, the correlation is smaller due to the number of small districts and parish between the largest urban centres. The correlation of incidence, population size, and urban/rural status is non-negligible (electronic supplementary material, figure S1 and table S1). This highlights the importance of controlling for these spatial features to assess differences in epidemic connectivity and infection dynamics between urban and rural districts. Districts tend to be closer to districts of the same type (e.g. rural districts are closer to other rural districts) and large urban districts are more likely to have large urban neighbours. Due to the importance of spatial proximity in regard to imported cases, it is necessary to isolate the effect of proximity from population size in order to further understand any potential urban and rural differences.

For example, examining districts within 150 km of London, we see a mean correlation in case reports of 0.48. Locations within 25 km of London have an average correlation of 0.67. Using a generalized linear model, we estimate that an increase of 10 km in distance is associated with a decrease in case correlation of −0.024, and an increase in population of 10 000 individuals is associated with an increase in correlation of 0.027 (details available in the electronic supplementary material, table S1). This means that 10 km of distance is comparable to a decrease in population of 10 000 people in terms of the mean correlation with London. Within 50 km of London there are 103 urban districts and only 21 rural districts. These results indicate the importance of controlling for the spatial influence of large cities when comparing urban and rural districts. Though we could attempt to investigate all locations in this way, it is a significant task to discover which large cities are influencing the epidemics for each district, particularly those in the hinterland, where many signals may mix and epidemics are comparatively rare [32].

### 4.2. Urban and rural district pairs: principal components analysis

**Figure 2.**
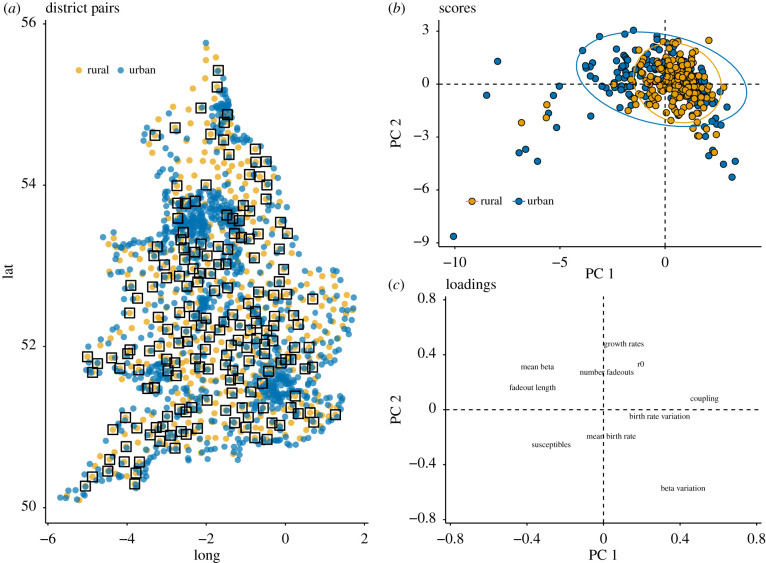
Principle components analysis on urban and rural district pairs. (*a*) The selected urban and rural districts, colour coded for urban/rural status with each pair joined in a box. We obtain pairs in a variety of spatial locations. (*b*) Projection on the first two principle components demonstrates some difference in the urban and rural districts. Though the scores follow the same general trend, rural areas have slightly less variation in their scores as shown by the 95% confidence ellipses. (*c*) The loadings of the first and second principle components show the parameters plotted at their scores on PC 1 (*x*-axis) and PC 2 (*y*-axis). The first principle component separates the districts based on one axis which correlates positively with epidemic coupling and negatively with fadeout length and seasonality in beta; the second component provides an axis with larger numbers of fadeouts weeks and high average transmission rates at one end, and low transmission rates and low transmission at the other. A full table of scores is included in the appendix.

When we subset the data to the selected paired districts (mapped in figure 2*a*) we observe a modest difference between urban and rural areas when applying principal components analysis to the estimated parameters. This decomposition shows that the two most variable axes of difference are (1) high coupling versus long fadeouts and (2) variation in seasonal transmission versus growth rates.

The first principle component (PC 1) accounts for 36% of the total variance in the data (electronic supplementary material, table S4). It is an axis which measures the data with coupling at one extreme (positive values) and fadeout length at the other (negative values). In other words, the spectrum which accounts for the most variance in the data is merely the separation of dynamics between locations which receive regular imports and demonstrate synchrony with the larger metapopulation, and isolated districts which experience long droughts of infection.

The second component (PC 2) explains 26% of the total variation in the data (electronic supplementary material, table S4). This component projects districts on an axis with variation in transmission (*β*) at one end (negative values) and growth rates on the other (positive values). In qualitative terms, this suggests that epidemics generally exhibit rapid epidemic spread or strong seasonal variation in transmission. In other words, locations with explosive epidemics tend to have less seasonal variation, implying outbreaks are more randomly spread throughout the year. Similarly, locations with more seasonal transmission experience epidemics which spread at a relatively slower pace. A district with a negative score on this axis will be characterized by high seasonality, indicating regular epidemics fed by relatively constant susceptible pools. A district with a positive score on the second component will likely have stochastic and explosive epidemics rather than annual or biennial school-based outbreaks (figure 2). We observe slight and statistically insignificant differences between urban and rural districts. On average, rural areas have slightly higher coefficients of coupling compared to urban areas. Conversely urban areas have on average fewer, more potent outbreaks.

With regard to where districts fall on PC 1 and PC 2, figure 2*b* shows districts either tend to have higher coupling estimates (positive on PC 1) accompanied by high variation in beta (negative on PC 2), or long fadeouts (negative on PC 1) and high growth rates (positive on PC 2). Therefore, if we interpret the two-dimensional space created by PC 1 and PC 2 we see that the majority of variance in the data can be described as a spectrum from areas with strong epidemic coupling and consistent seasonality and one extreme and infrequent violent epidemics at the other extreme. This is consistent with previous studies of large and small urban locations [5,21,22].

The space created by the first and second principal components has a plausible association with population size. We expect large places to receive more import cases and thus to have larger coupling estimates and shorter time between epidemics. We also expect large places to have more regularity in seasonal transmission as dictated by the school calendar, while smaller places are more vulnerable to random outbreaks. We also see that the largest outliers in the data (third quadrant) have long fadeouts and high susceptible population remaining after each epidemic. This suggests that a few locations in the data have very long interepidemic periods with few outbreaks which are not sufficient in size to diminish the susceptible population. This is consistent with previous studies of measles dynamics in E&W [5,21,22].

**Figure 3.**
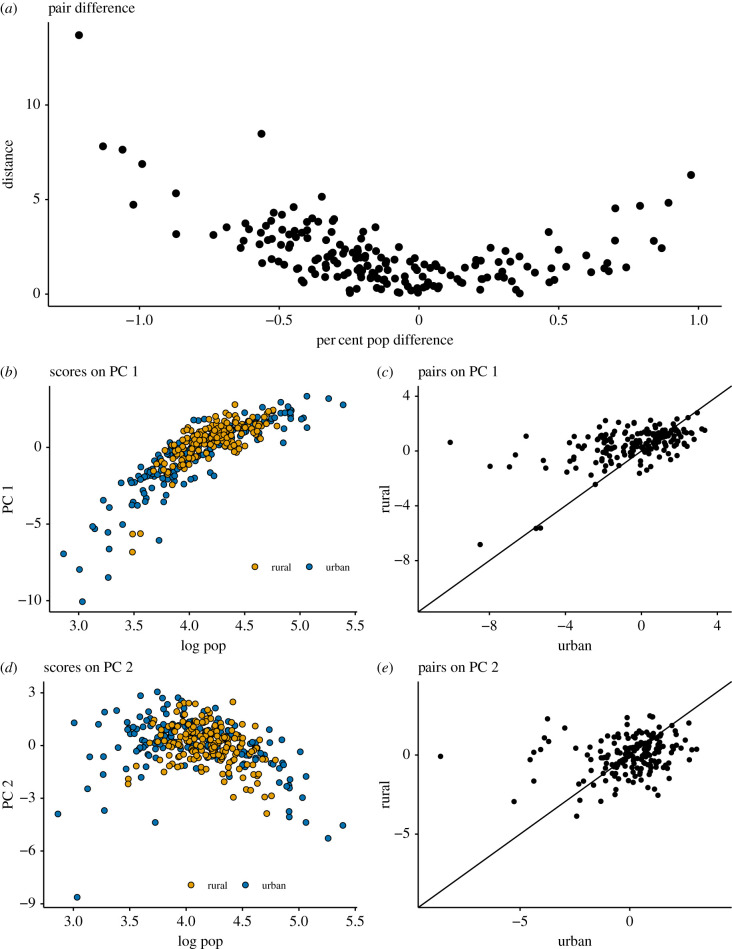
Untangling space and size in urban and rural districts. (*a*) The euclidean distance between pairs when projected on the first and second principle components as a function of the per cent difference in population size. (*b*,*d*) The relationship between population size and scores on each of the components. Panels (*c*,*e*) plot urban and rural district pairs against each other on the basis of their scores on the first (*c*) and second (*e*) principle components. Scores on or near the plotted line of identity indicate matching scores for the pairs. The score of an urban district has little to no relationship with the score of it is rural counterpart in general (*c*,*e*). However, as shown by (*a*) we see when pairs are of comparable size, they tend to have similar scores. If space had been the primary driver of epidemic dynamics, we would expect the points in figures *c* and *e* to follow the identity line. If pronounced differences existed between urban and rural locations, we would not expect pairs to look like each other or be well predicted by population size alone. The projection of the pairs on the first two components appears to be well determined by population size (*b*,*d*) and the distance between each pair and its neighbour is well determined by the per cent difference in their populations. This would imply that spatial location has a marginal impact on epidemic dynamics and population size is a stronger driver of dynamics than urban or rural status.

Principle component results demonstrate no statistically significant difference between urban and rural areas. When comparing adjacent districts we see district neighbours do not resemble each other (figure 3*c*,*e*) and that population is the main driver of differences both in terms of raw projections (figure 3*b*,*d*) as well as in determining the difference between urban and rural pairs (figure 3*a*). We classify the difference between pairs as the euclidean distance between the district’s scores on the first and second principal components. We see that this difference is well explained by the per cent difference in population size. This suggests size, rather than space is the predominant driver of variation in disease dynamics.

Though urban and rural areas may not differ systematically, the analysis to this point does not describe how neighbouring towns and cities interact with each other. Although pairs do not demonstrate coherence in their scores on PC 1 and PC 2, we know infections move through space and expect to see some evidence of epidemic interactions between neighbours. In order to investigate these district pairs in more detail, we examine the case data directly.

### 4.3. Urban and rural district pairs: epidemic exchange

**Figure 4.**
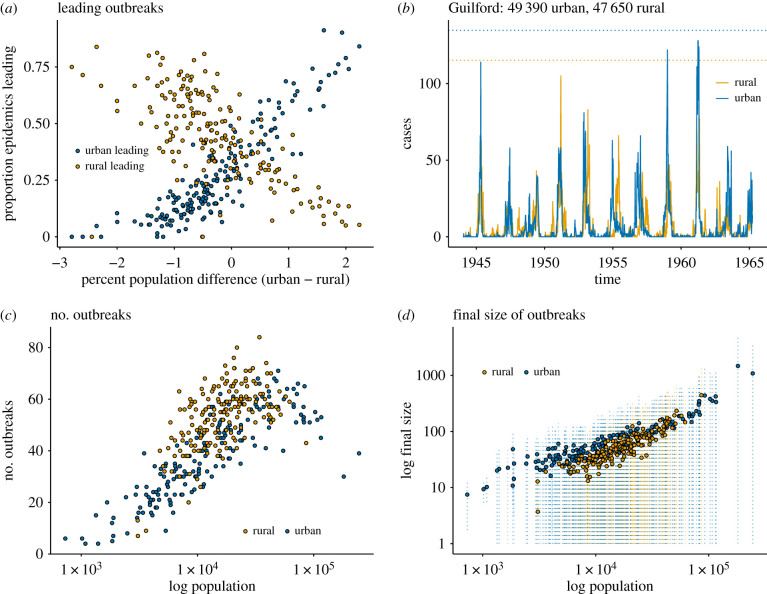
Epidemic interactions among neighbouring urban and rural districts. (*a*) The per cent of the population difference (relative to the urban district) compared to the proportion of epidemics led by urban or rural district. We see for both rural and urban areas, the per cent of epidemics led increases as the per cent difference in size increases. This confirms previous findings that large areas provide epidemic spillover to proximate smaller areas, this highlights the cascading effect of epidemics at a local level. (*b*) Guilford provides an example of this subtle dynamics wherein an urban area has fewer small outbreaks and larger major epidemics even when the neighbours are of similar sizes. The dotted lines indicate the average final outbreak size (134 total average infections for the urban district and 115 for the rural). In the case of Guilford, both the urban and rural district have 57 outbreaks, the rural district has 13 larger outbreaks while the urban district has 11. These 13 larger rural outbreaks are smaller in terms of final size than the 11 outbreaks in the urban district. (*c*) Rural areas have more outbreaks than urban areas of comparable size. (*d*) Mean final size of outbreaks for urban and rural areas (log scale) with standard errors. Rural areas tend to have smaller outbreaks on average, though the standard errors are large and the difference is not statistically significant.

Projections on the first component demonstrate that urban areas may have longer fadeouts and lower estimates of coupling than rural neighbours. Since uncertainty around estimates of coupling can be large, particularly for larger districts, we examine the differences between urban and rural epidemics directly from the case data. Consistent with the dynamics suggested by the results of PCA, we find that rural areas fade out less and for shorter periods of time, resulting in more frequent, smaller epidemics (figure 4*c*,*d*). By contrast, urban areas are characterized by more regular short epidemics (figure 4*c*,*d*). Urban and rural areas do not differ in the number of large (final number of infections greater than the mean) outbreaks, which increases consistently with population size for both district types (electronic supplementary material, figure S4(B)).

We additionally find that larger districts tend to lead the epidemics of their smaller neighbours. The relationship between the difference in population and the proportion of epidemics lead in each location is strong (figure 4*a*). Larger places appear to act as a importer of cases to their smaller neighbours, mirroring national patterns of epidemic spillover at a small scale. An example of such a pair can be seen in figure 4*b*. This suggests transmission cascades from larger places to smaller places in concurrence with previous findings [39], but replicated at a local scale. We do find a nominal, though significant difference between urban and rural areas particularly at smaller sizes. Urban areas have fewer, larger outbreaks when compared to rural areas of comparable size (figure 4*c*,*d*). This difference is slight though statistically significant if we look exclusively at small areas. Large urban and rural areas do not demonstrate a statistically significant difference. This indicates small urban populations may be nominally more well mixed than small rural populations. Though this may seem intuitive and obvious it is important to keep in mind the crucial role of schools in measles dynamics. The population mixing rates relevant to this system are those of school-age children. These results therefore indicate that urban schoolchildren may be better mixed, with more cross-school mixing, than rural counterparts. Alternatively urban areas may have fewer schools compared with rural counterparts, creating more concentrated contagion hotspots relative to rural districts.

As a robustness check, we compare these same measures using population density for the subset of districts for which we have estimates. We see that differences in population density correspond well to final size estimates, but do not explain epidemic leads and lags (electronic supplementary material, figure S7). In particular, we see that denser areas generally have larger and fewer outbreaks, while less dense locations have more, smaller outbreaks. Additionally, relative density shows no correlation with epidemic leads or lags. Though these results represent only a subset of the paired data, they increase our confidence in the veracity of the small differences we observe.

## 5. Discussion

Understanding how transmission may vary between rural (or sparsely populated) and urban (or densely populated) areas is a critical area of research in a rapidly urbanizing world. The United Nations predicts that nearly 70% of the global population will live in urban areas by 2050. Though previous analysis on this unique detailed dataset has suggested measles transmission is size and density-independent with a strong seasonality in transmission and signature of contagion movement between locations [26], the urgency of contemporary changes necessitates a more complete understanding of potential differences across settings. Previous analyses have been limited to urban areas. Expanding this to include rural areas provides a more complete understanding of metapopulation dynamics and variation across space and urban/rural district type. The complete and rich nature of this dataset make it uniquely suited to be an initial case study for such investigations.

This analysis shows that infectious dynamics are not uniform across locations. However, while we find an inverse relationship between infectious disease fadeouts and coupling of locations to the larger metapopulation, and between epidemic growth rates and seasonality, urban and rural locations follow the same pattern in spite of potential structural differences. Although we see a slight difference on average between urban and rural areas when controlling for location and population, the overall patterns are consistent. Population size is the most significant driver of epidemic dynamics (though total number of births is a comparable predictor and highly correlated with population size). Additionally, while location does appear to impact dynamics, the similarity by pairs is not what we would expect if differences were entirely spatial (figure 3). The difference in population sizes appears to explain many of the differences we observe. These findings are generally consistent with Ferrari *et al.*’s results for Nigerian measles epidemics [16]. Ferrari *et al.* find a rural/urban gradient characterized by reduced seasonal amplitude in sparsely populated settings as well as climatically driven contact rates. Cross-location contact rates are even more sporadic in the Nigerian context, indicating that much of the consistency between urban/rural locations in E&W is likely driven by a consistent seasonal forcing mechanism (school calendar) as well as more frequent cross-location mixing.

In addition to confirming similarity between urban and rural areas, principal components analysis shows an important difference in large (above 10 000) and small (below 10 000) populations. Larger places can be characterized by more frequent epidemics with a typical seasonal signature, while small places are characterized by stochastic epidemics which are slower and do not deplete susceptible populations. This confirms that small places inherit epidemics as spillover from bigger neighbours (figure 3). When we investigate epidemic interactions between urban and rural areas we find size is the most important when determining which location will kick off a local epidemic (figure 4). In other words, we see large scale metapopulation dynamics mirrored in these urban and rural pairs. The larger member of each pair seems to serve as an epidemic feeder for its smaller neighbour. In this case, it does appear to be size which drives the influx of cases rather than urban/rural status. The differences are most profound when population sizes are substantially divergent (on the order of 100–300%). When urban and rural neighbours are of comparable size, there is no clear epidemic leader (figure 4).

On average, urban epidemics are contained within (in a temporal sense) their rural neighbour epidemics, 23% of the time; rural epidemics are contained within urban epidemics only 14% of the time on average. If we examine pairs for whom the urban location is approximately twice the size of the rural location, we see that 54% of rural outbreaks are contained within the larger urban outbreak. If we examine the converse, when urban areas are half the size of their rural neighbours, we see that 37% of outbreaks in these small urban centres are contained within those of their rural neighbours.

Urban districts have 38 individual outbreaks on average, while rural districts have approximately 53. Urban districts have about 5.5 large epidemics on average and rural areas have 7. In terms of final size, urban outbreaks result in 96 infections on average, while rural outbreaks have a mean number of infections of 61. In summary, urban districts have fewer outbreaks which infect a greater number of residents compared to their rural neighbours. These numbers correspond the slight differences we see in figure 4*c*,*d*.

Urban districts lead their rural counterparts 27% of the time and rural districts lead urban neighbours 44% of the time. The lag time between urban and rural neighbours on average is near zero (−0.2 for urban and 0.18 for rural). Examining urban districts that are twice the size of rural neighbours, urban districts lead epidemics by about two weeks and lead 67% of epidemics. Analogously, rural districts that are twice as large as their rural counterparts lead by about 1.3 weeks on average and lead epidemics 57% of the time. This indicates the importance of relative population size in a competing destination's framework of receiving imported cases. It may indicate that the increase in receiving cases as a result of increasing population size is greater for urban areas than for rural areas. In other words, larger urban areas are marginally more attractive for cases than rural counterparts. It is also sometimes the case that rural districts completely or mostly surround urban neighbours, for these districts early rural outbreaks may be the result of incidental rural infection en route to urban districts. However, more explicit data on population movement patterns, particularly those of children, are necessary to verify this argument. It is also possible that epidemics in urban districts create multiple rural echos, producing multiple smaller epidemics.

Controlling for population size, we observe a small though statistical significant difference in epidemic behaviour between urban and rural districts (electronic supplementary material, figure S6). Rural areas have more frequent, smaller outbreaks while urban neighbours have relatively fewer, larger epidemics. This confirms previous findings that aggregated urban dynamics showed more intense epidemics relative to aggregated rural data [28]. Here, we have explicitly shown this behaviour at the district level, controlling for space and proximity to large cities.

**Figure 5.**
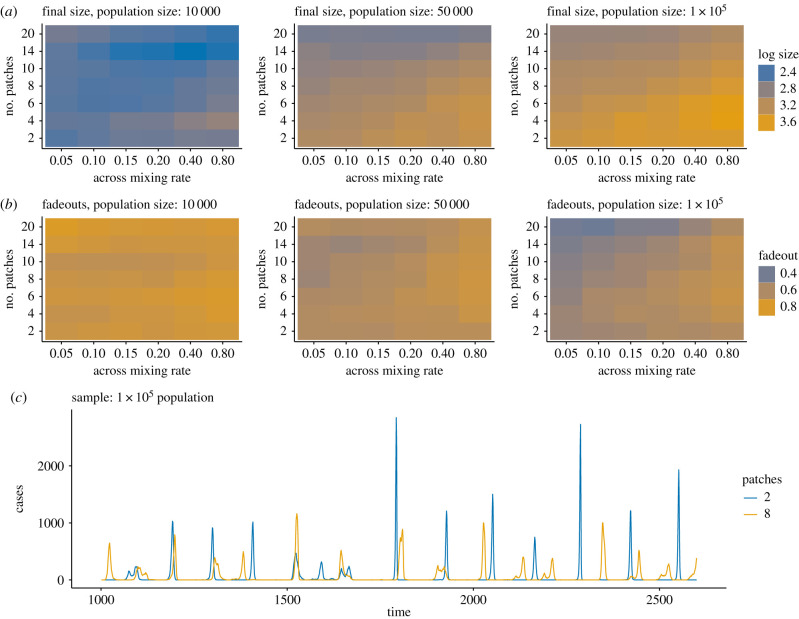
Simulations showing the impact of patch mixing patterns on fadeouts and outbreak size for hypothetical towns with populations of 10 000, 50 000 and 100 000. In (*a*), each tile plot shows mean final sizes of outbreaks for a given mixing rate across patches (across) and number of patches (patches) for a single population size. (*b*) The proportion fadeout for each combination of mixing rates and patches. The across patch mixing rates were reduced such that they represented a fraction (given by the ‘across’ axis of the internal mixing rate. (*c*) Example time series for a population of 100 000 with an across patch mixing rate of 20% of the within patch mixing rate. Cases are shown for sample simulations with two and eight patches. Within patch mixing rates were held constant to produce an *R*_0_ of 20, a typical estimate for measles. Simulations show the number of outbreaks increases as the number of patches increases (*b*) and the final size of the outbreaks decreases as the number of patches increases (*a*). In addition, as mixing rates between patches increase, the proportion fadeout biweeks increases and the final size increases. This supports the conclusion that rural areas may be characterized by patch mixing with relatively weak mixing between leading to more, smaller outbreaks when compared with urban counterparts.

To verify this behaviour with known population densities and mixing rates, we simulate epidemics in a number of communities. We vary the total size, number of patches (as a proxy for multiple infection hotspots), and mixing rates between patches. We then examine fadeout proportions and final sizes across a combination of patch numbers and mixing rates for each community size (figure 5). We assume the population and birth rates within each patch are equal and the within-patch transmission rate is constant for an *R*_0_ of 20, a reasonable estimate for measles [27]. Across-patch transmission rate is fixed at 1%, 5%, 10%, 15%, 20%, 40% and 80% of the within-patch rate. From the simulated incidence data, we calculate the average number of fadeouts and the average final size of outbreaks. As expected, increasing patches reduces the final size of each epidemic and reduces the fadeout proportions. Additionally, increasing the across-patch mixing rate increases the final size and increases the proportion of fadeouts. This is congruent with the slight difference we observe between small urban and rural districts.

Heterogeneous contact patterns in rural areas may be driving these subtle differences relative to urban areas which may be closer to well mixed. Rural districts may have several transmission hotspots (schools) distributed over a greater area. Strong within-school mixing coupled with weak across school mixing rates would result in multiple small outbreaks. By contrast, a denser urban area will provide more opportunities for mixing even when multiple schools exist leading to fewer, larger outbreaks, as demonstrated by the simulation results in figure 5.

## 6. Future work

School-level data are crucial for further disentangling differences between urban and rural areas. Specifically, school data could elucidate whether rural areas are receiving more cases than urban areas or if multiple hotspots are producing multiple epidemics from within the community. However, to our knowledge adequate data for this time period does not exist in an appropriate scale to address this question. Furthermore, even if we optimistically assume urban and rural designations are substantive in these data, it is also true that urban and rural distinction in E&W is not comparable to global differences in urban and rural environments. That is, rural areas are denser than global rural extremes and urban areas are smaller and less dense than contemporary megacities. Despite this, our exhaustive analysis of urban and rural disease dynamics in this detailed dataset provides a strong first examination of possible differences.

An additional challenge in this context is the age profile of the susceptible class (typically schoolchildren, aged between 5 and 10 years), as well as the primary transmission location (schools). The movement pattern of schoolchildren is unlikely to exhibit as much variation in mobility or contact rates across contexts; this may be especially true in E&W during this time period where school attendance is compulsory for young children. As the majority of contacts for this age group occur in school settings, there is likely not as much variability in these contact rates in urban versus rural settings relative to other types of contact.

Extrapolation of these findings to other contexts is limited to acute immunizing pathogens in countries of similar levels of development. However, we would expect the differences to be more pronounced in countries with more variation in urban/rural settings. Though it is likely that the districts in this dataset do not adequately reflect urban/rural differences in other countries, the methods in this paper may serve as a useful framework for urban/rural analysis in other contexts.

In the wake of contemporary measles outbreaks and declining vaccine coverage, comprehension of measles transmission has gained renewed urgency. Understanding transmission over metapopulation structures is vital for predicting outbreaks and planning interventions. Additionally, understanding the spread of disease over different population densities and mixing patterns is crucial in a rapidly urbanizing world. This analysis illustrates the cascading of disease transmission even at local levels, suggesting the larger of two populations is at greater risk of infection holding geographical location relatively constant. Furthermore, it suggests transmission may be slightly more rapid in dense areas but that persistence may be greater in sparse areas. The strength of transmission across locations highlights the potency of measles infection across scales. In addition, case data demonstrates that infections cascade from endemic areas to places of next-largest size, and that this pattern persists even at extremely local scales. In general, this suggests the importance of targeting interventions in large population centres were disease outbreaks can grow to epidemic levels and instituting control strategies to prevent disease from travelling to subsequent locations. Finally, results on rural transmission highlight the importance of understanding local population mixing patterns and maintaining records on the number and spatial distribution of community hotspots.

Further research is necessary to build a comprehensive understanding of transmission in urban and rural areas. In particular, more detailed data on population densities within urban/rural areas as well as mixing patterns will be critical in untangling the pace and persistence of epidemics. In particular, similar studies in contexts with greater variation in urban and rural settings could help elucidate the impact of density on mixing rates for this particular susceptible class. In addition to highlighting slight differences between urban and rural districts within a metapopulation, this work demonstrates the importance of the spatial scale of reporting for estimates of disease transmission. Aggregating several transmission zones into one reporting region may reduce estimates of contagion and overestimate import rates.

## Supplementary Material

Supplement

## Supplementary Material

Area

## Supplementary Material

Measles Data; Code

## Data accessibility

The data and code necessary to replicate the findings presented in this paper have been included as electronic supplementary material.
