# Supplementary material for: Structure, space and size: competing drivers of variation in urban and rural measles transmission
Source: J R Soc Interface. 2020 Jul 8;17(168):20200010. doi: 10.1098/rsif.2020.0010 (PMC7423418; doi:10.1098/rsif.2020.0010)
Supplement: Supplement [file rsif20200010supp1.pdf]

Structure, Space, and Size: Competing Drivers of Variation in Urban and Rural Measles Transmission: Supplementary Material

Hannah M. Korevaar, Bryan T. Grenfell, C. Jessica Metcalf

Spatial Patterns of Disease and Population

To illustrate the importance of spatial confounding effects, we calculate the correlogram of the incidence (in space-time), population size and urban/rural district type (in space). We also plot the temporal correlation of incidence data between London and districts within 150 km of London. We see that correlation decreases with distance and that larger districts have higher correlations with London controlling for distance. We also see that there are more urban districts in the immediate periphery of London. This demonstrates the importance of controlling for space when investigating urban and rural disease dynamics.

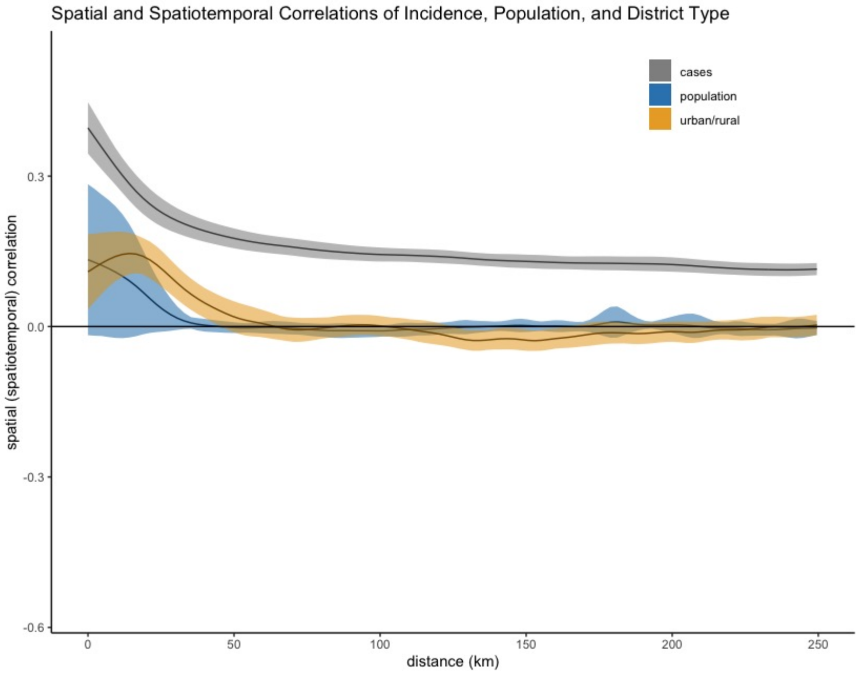

Figure 1: Spatial correlation of population size, urban/rural district type and the spatiotemporal correlation of incidence. We see the high spatiotemporal correlation of measles cases among near neighbors (grey, top line) which drops off with increasing distance. By 50 km, it has reached the mean temporal correlation of .14. The spatial correlation of population size and district type is also somewhat correlated among nearest neighbors and drops off to statistically zero after 50 km of distance.

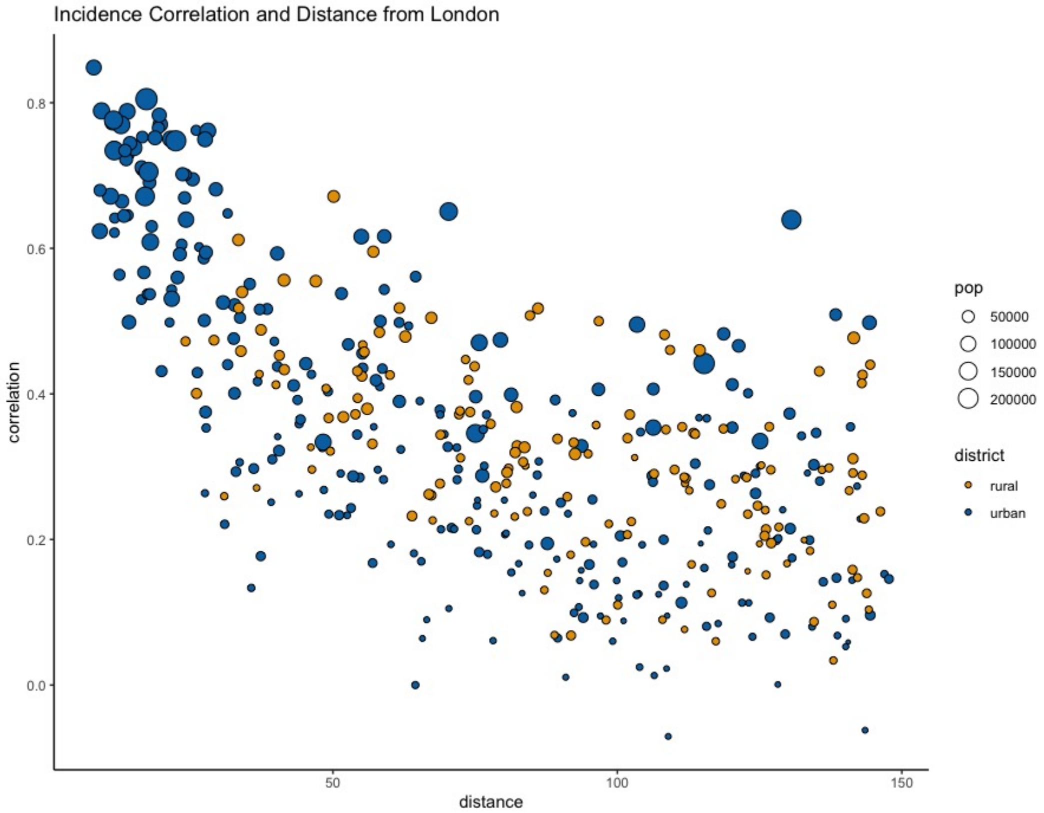

Figure 2: Incidence correlation decreases with increased distance from London. Population size increases correlation with London dynamics controlling for distance. GLM regression results indicate that correlation decreases, on average -0.0023 per kilometer and increases .0026 per 1000 people.

Table 1: GLM results for districts within 150 km of London, regressing case correlation on distance, population size, and urban/rural designation.

	Dependent variable:
	cor
distance (km)	$-2.387e$-03***
	$(1.506e-04)$
population	$2.663e$-06***
	$(1.687e-07)$
urban district	$-4.687e$-02***
	(0.012)
constant	0.481***
	(0.018)
Observations	424
Log Likelihood	330.225
Akaike Inf. Crit.	−652.450
Note:	*p<0.1; **p<0.05; ***p<0.01

TSIR

As a robustness check, we profiled each location across all fitting options available in the tsiR package. Specifically, we fit tsiR using every reasonable combination of regression type for susceptible reconstruction, switching the axes for cumulative births and cases, as well as the generalized linear model family used to estimate parameters. We then select the model with the best likelihood score and use those parameter estimates. This produces slightly different parameter estimates for each location, but the comparative results are consistent. In general we observe a slightly attenuated correlation with population size for our aggregate measures, this is consistent with our belief that there may be a slight bias in small populations. This will be examined more thoroughly in upcoming work. When we compare the district pairs, the shape of the curve in Figure 3 which shows the difference of pairs by population difference is virtually the same even when the association with population size is attenuated in the "robust" estimates. Regression results (discussed later in the supplement) demonstrating the relative importance of population size also hold. As the results did not change, we opt to present the simpler fitting options in order to be consistent with previous analyses.

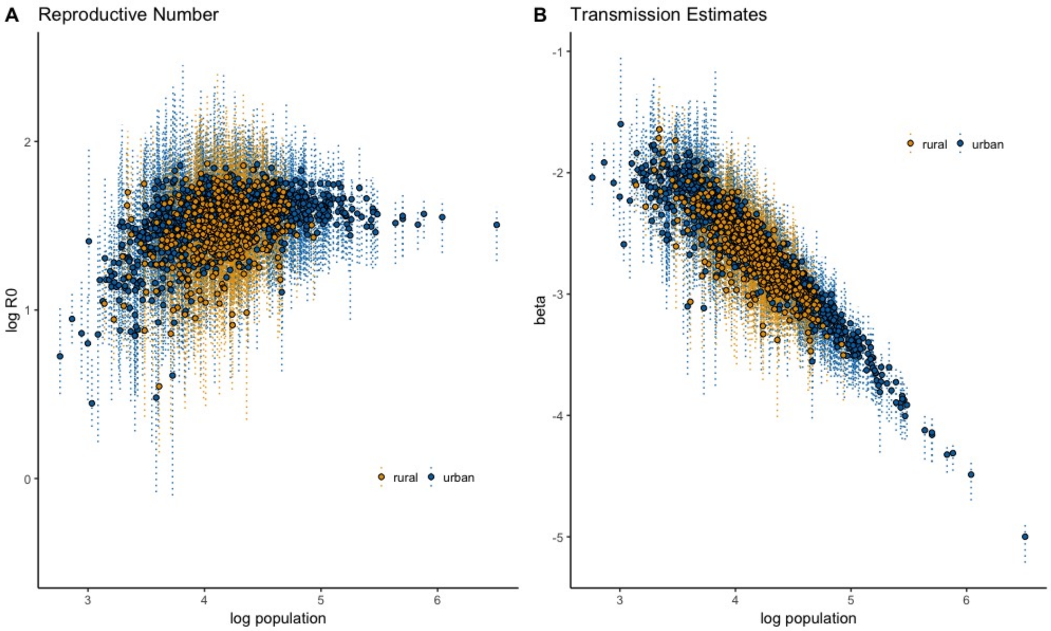

Figure 3: Estimates of the basic reproductive number by population. Here we see some evidence of potential downward bias in the small populations. Though this trend holds even when parameters from optimized regression selections are used. Future work will examine the potential contributions of modeling bias, but we expect they are quite small.

Parameter	Mean	Median	Standard Deviation
Mean β (urban)	0.00339	0.00237	0.00311
Mean β (rural)	0.00274	0.00214	0.00234
R_0 (urban)	36.3	36.8	11.8
R_0 (rural)	33.7	32.8	11.7
R^2 for short term prediction (urban)	.9988	.9994	.0015
R^2 for short term prediction (rural)	.9989	.9993	.0013

Table 2: Parameter estimates and model fit for TSIR modelling process. Here we report mean, median and standard deviation values for our estimates of transmission (β) as well as the corresponding estimates for R_0 across all districts, separated by urban/rural designation. We also report the R^2 for step-ahead predictions using the fitted TSIR parameters. The predicted case numbers consistently fit the observed cases well across districts.

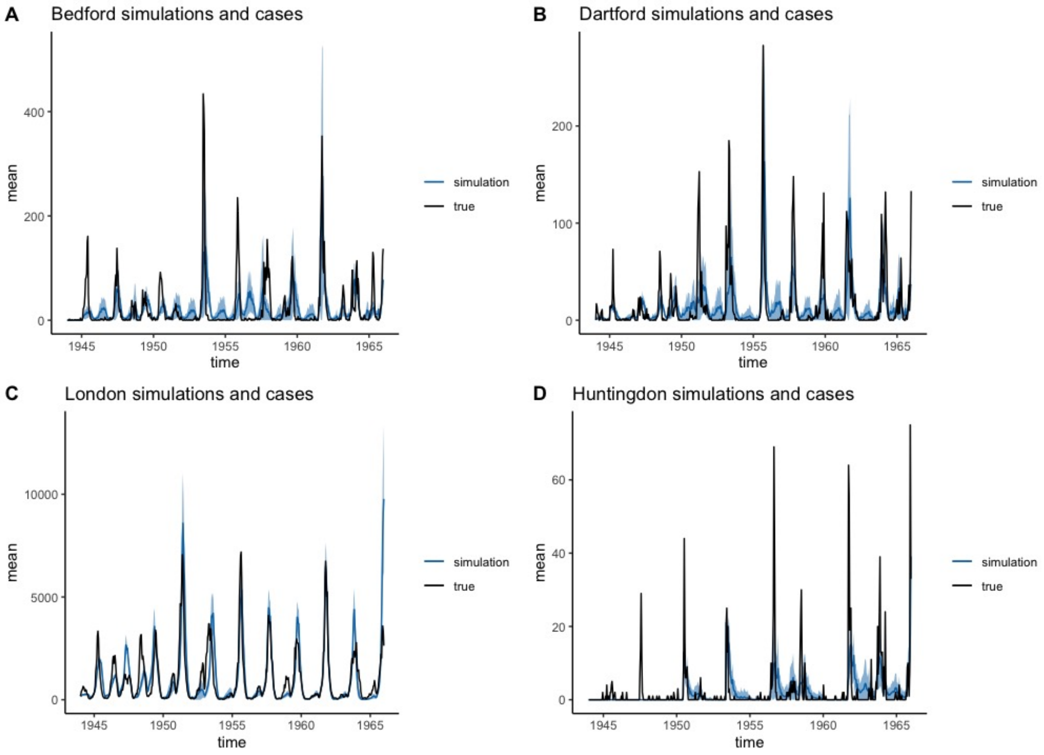

Figure 4: A comparison of TSIR-based simulations using estimated parameters on four locations in the data set. True cases plotted in black, mean simulations plotted in blue with ribbons showing standard deviations of simulations. This demonstrates the suitability of parameter estimates across a variety of locations; from the largest place (C) London (population approximately 3 million), to Bedford (panel A, population 57,000), Dartford (panel B, population 41,000), and Huntingdon (panel D, population 6,400).

Pair selection

Urban and rural pairs were selected by virtue of having the same name. Each represents and urban district and a corresponding rural area. We then confirm that each rural district neighbors the urban district with the same name. In total we have 179 districts of various sizes in a variety of locations.

Districts	Median Population	Mean Population	Minimum Population	Maximum Population	Standard Deviation
Urban	11411	23472	720	245236	32047
Rural	16552	19433	3060	8608	12600

Table 3: Paired districts: descriptive demographic statistics.

PCA

Table 4: Variable loadings for each of the principal components and the amount of variance each component explains. Bold numbers indicate the covariates we use to describe the component axes in the main text. These values were selected due to their high loadings relative to other covariates. Additionally each variable has a high loading on either the first or the second principal component, but not both. This makes these variables specific to each component and therefore good descriptors of each axis.

variable	PC 1	PC 2	PC 3	PC 4	PC 5	PC 6	PC 7	PC 8	PC 9	PC 10
coupling	**0.488**	-0.069	0.165	-0.067	-0.075	0.121	0.170	-0.037	0.520	0.637
susceptibles	-0.307	-0.372	-0.350	-0.210	-0.047	0.057	0.169	0.692	0.296	0
growth rates	0.025	**0.539**	-0.305	0.106	0.109	0.720	-0.214	0.157	0.051	0.030
fadeout length	**-0.489**	-0.012	0.114	0.212	0.133	0.026	0.061	0.070	-0.419	0.707
birth rates	0.207	-0.110	-0.233	0.742	-0.554	-0.063	0.031	0.140	-0.093	-0.009
β variation	0.254	**-0.453**	0.294	-0.125	-0.066	0.257	-0.651	0.233	-0.281	0.028
β	-0.336	0.291	0.369	0.235	-0.056	-0.294	-0.477	0.185	0.509	-0.011
birth variation	0.283	-0.134	-0.282	0.353	0.765	-0.272	-0.155	0.110	0.047	0.030
number fadeouts	0.218	0.378	-0.390	-0.385	-0.228	-0.479	-0.274	0.173	-0.228	0.267
R_0	0.284	0.322	0.486	0.036	0.106	-0.058	0.376	0.582	-0.250	-0.144
SD	35.5	25.8	21.7	18.5	15.5	13.8	12.3	11.1	7.8	5.3
Prop	0.356	0.188	0.133	0.097	0.068	0.054	0.043	0.035	0.017	0.008
Cum	0.356	0.544	0.677	0.774	0.842	0.896	0.940	0.975	0.992	1

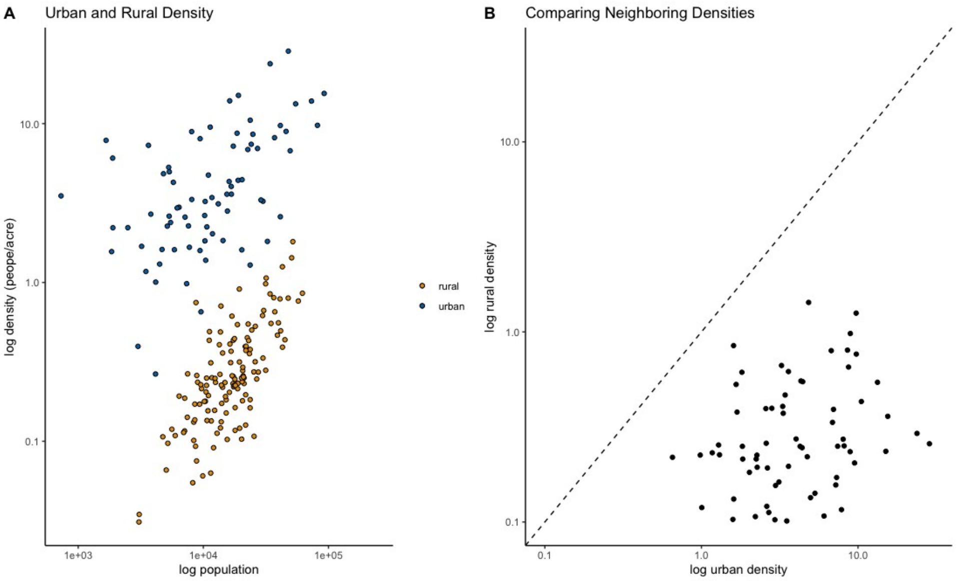

Figure 5: Density estimates in people per acre for the 78 urban areas and 136 rural areas for which we obtained land area estimates. We see that rural districts are consistently less dense than urban districts, reducing the possibility of misclassification bias in our paired data.

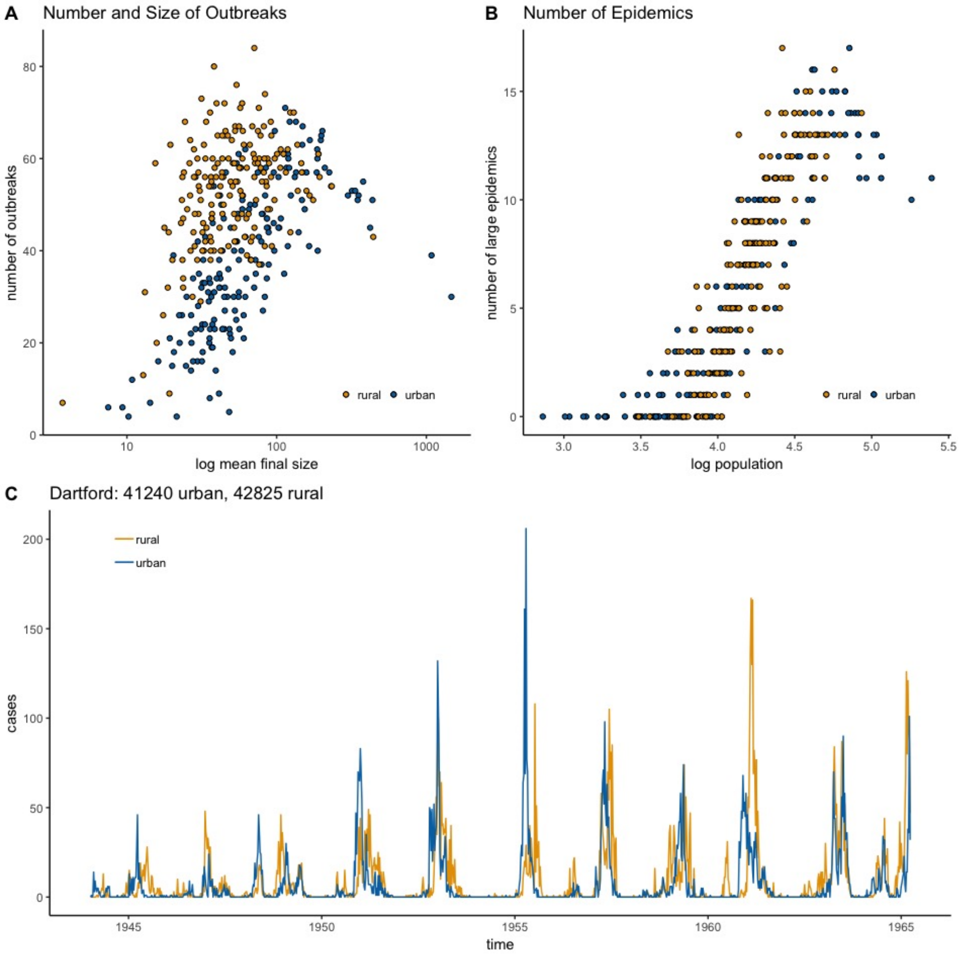

Figure 6: Additional figures demonstrating (A) the difference in size and number of epidemics between urban and rural districts and (B) the consistency in the number of large outbreaks (¿ 14 weeks). Another example of an urban and rural pair which demonstrate the subtle difference even when population sizes are comparable.

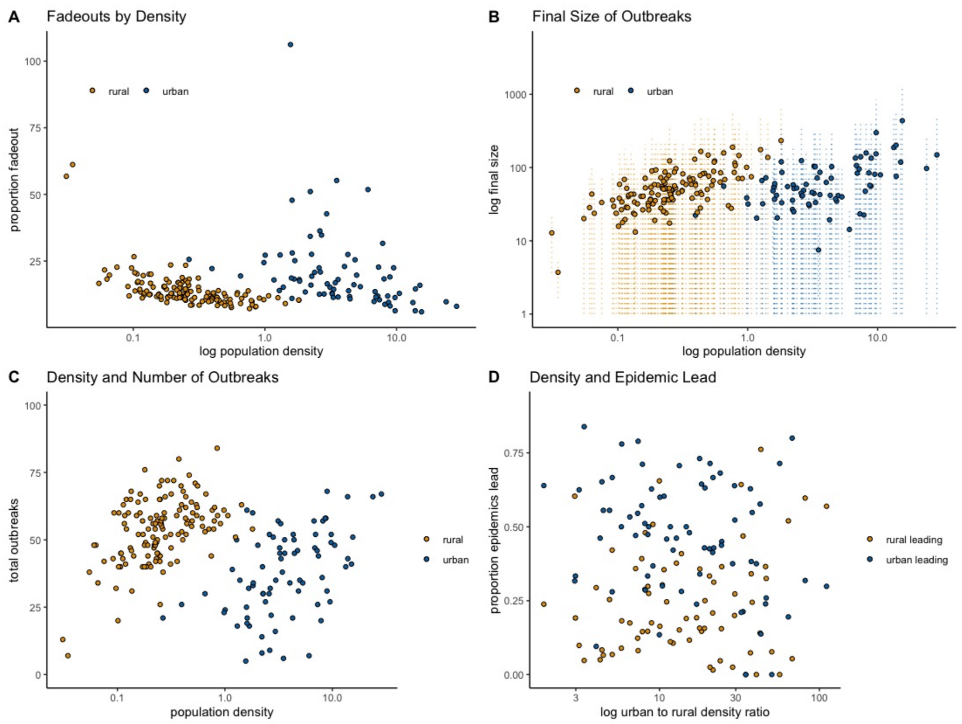

Figure 7: Population density may explain (A) differences in the proportion of fadeouts across locations and (B) the final size of epidemics. We see some evidence (C) that the total number of outbreaks is higher in less dense areas. Density does little to determine (D) which district (urban or rural) leads each local epidemic. This reinforces our hypothesis that population size determines which location is likely to receive new imported cases and population density explains local dynamics once an outbreaks has been started. In particular, dense areas will have fewer, larger outbreaks relative to less dense counterparts.

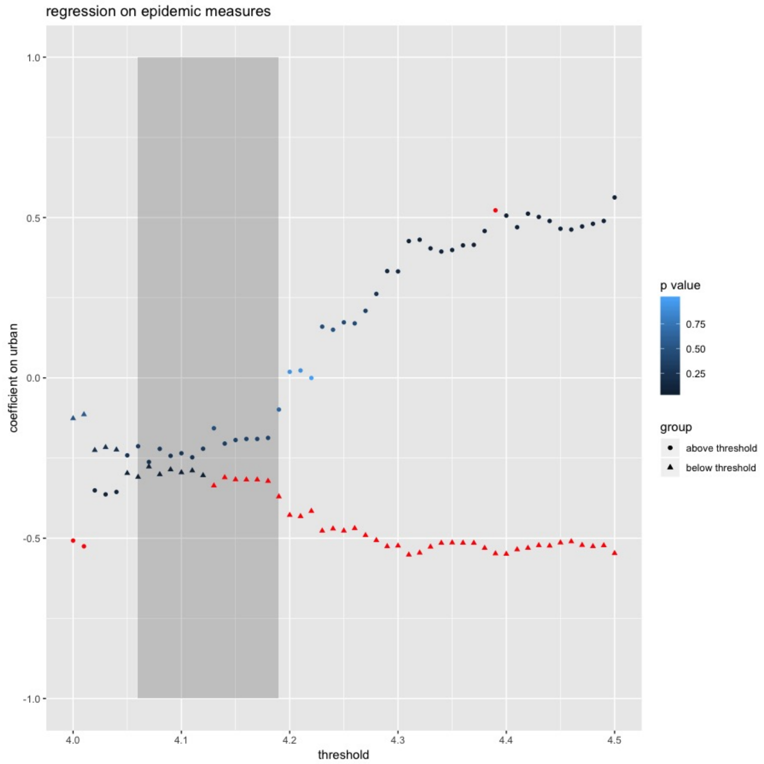

Figure 8: Size and significance of the coefficient urban/rural dummy when different thresholds are used for large/small populations. These are the coefficients calculated for the epidemic parameter data. The shaded region demonstrates the area where the adjusted R-squared was highest for the regression. Red points indicate statistical significance at the .05 level. We see some statistical significance at thresholds above .415, but the magnitude of these coefficients is consistently near 0.

Pair Regression

We tested the association of urban and rural designation as well as population size using linear regression. As the shape of the association with population size is demonstrably non-linear (Figure **??**), we allowed for different intercepts and slopes for small (log population ¡ 4.15) and large populations (log population ¿ 4.15). This threshold was the most generous in terms of finding an effect of urban/rural designation and maximizing the adjusted r-squared of the regression. Even with the most generous threshold, the association of urban/rural designation is statistically significant only for small locations, with the effect size representing approximately one tenth that of log population. In other words, the marginal difference between urban and rural locations is comparable to a .1% change in population size. We believe this does not constitute a substantive difference. Even still this represents only a difference on the first principal component which explains only 39% of the total variance.

Since the data is locally linear with population size, we split the data above and below around 10^4. We test sensitivity to this division by moving the threshold from 10^4 to $10^{4.5}$ by .01 and rerunning the regression. The statistical significance of the coefficient on urban is sensitive to the high/low threshold. When we select the threshold which maximizes the adjusted r-squared, the coefficient on urban status is significant for small places but not for large places is and is comparable to one tenth that on log population. In other words, for small places a shift from rural to urban is equivalent to a .1% increase in population size. Therefore, though the results are statistically significant, the size of the association is inconsequential. We further test these results by comparing with a regression on the spectral density results. The difference between urban and rural districts on their components is of a comparable size as that of the qualitative measures, however, the statistical significance is diminished.
